# Correction: Forecasting the rheological state properties of self-compacting concrete mixes using the response surface methodology technique for sustainable structural concreting

**DOI:** 10.1371/journal.pone.0307891

**Published:** 2024-07-23

**Authors:** Edwin Zumba, Nancy Velasco, Edison Marcelo Melendres Medina, Jorge Buñay, Nestor Augusto Estrada Brito, Kennedy C. Onyelowe, Nakkeeran Ganasen, Shadi Hanandeh

The fourth author’s name is spelled incorrectly. The correct name is Jorge Buñay.

The correct citation is: Zumba E, Velasco N, Melendres Medina EM, Buñay J, Estrada Brito NA, Onyelowe KC, et al. (2024) Forecasting the rheological state properties of self-compacting concrete mixes using the response surface methodology technique for sustainable structural concreting. PLOS ONE 19(7): e0302202. https://doi.org/10.1371/journal.pone.0302202.

There are typographical errors in the affiliations. The correct affiliations are as follows:

Edwin Zumba^1,2^, Nancy Velasco^3^, Edison Marcelo Melendres Medina^4^, Jorge Buñay^5^, Nestor Augusto Estrada Brito^3^, Kennedy C. Onyelowe^6,7^, Nakkeeran Ganasen^8^, Shadi Hanandeh^9^

**1** Universidad Nacional de Chimborazo (UNACH), Riobamba, Ecuador, **2** Universidad Politécnica de Madrid (UPM), Madrid, Spain, **3** Facultad de Informática y Electrónica, Escuela Superior Politécnica de Chimborazo (ESPOCH), Riobamba, Chimborazo, Ecuador, **4** Facultad de Administración de Empresas, Escuela Superior Politécnica de Chimborazo (ESPOCH), Riobamba, Chimborazo, Ecuador, **5** Facultad de Mecánica, Escuela Superior Politécnica de Chimborazo (ESPOCH), Riobamba, Chimborazo, Ecuador, **6** Department of Civil Engineering, Michael Okpara University of Agriculture, Umudike, Nigeria, **7** Department of Civil Engineering, Kampala International University, Kampala, Uganda, **8** Department of Civil Engineering, SRM Institute of Science and Technology, Tamil Nadu, India, **9** Department of Civil Engineering, Al-Balqa Applied University, As-Salt, Jordan.
